# Supplementary material for: Supplemented Infant Formula and Human Breast Milk Show Similar Patterns in Modulating Infant Microbiota Composition and Function In Vitro
Source: Int J Mol Sci. 2024 Feb 2;25(3):1806. doi: 10.3390/ijms25031806 (PMC10855883; doi:10.3390/ijms25031806)
Supplement: Supplementary file 1 [file ijms-25-01806-s001.zip › ijms-2778974-supplementary.pdf]

International Journal of *Molecular Sciences*

Supplementary Table S1. Average relative abundance of genera in each treatment at each timepoint and the prevalence of the genus in the sample pool (N=36)

Genus	TEST FORMULA (RA)				BASE FORMULA (RA)				BREAS TMILK (RA)				Positive samples (n; N=36)	Positive Samples (%)
	T=0h	T=6h	T-24h	T=48h	T=0h	T=6h	T-24h	T=48h	T=0h	T=6h	T-24h	T=48h		
Streptococcus	0,3788	0,6373	0,6351	0,0368	0,2103	0,4586	0,5151	0,4799	0,3865	0,6775	0,6233	0,0465	36	100
Raoultella	0,2119	0,0680	0,0487	0,0351	0,2723	0,1695	0,1152	0,1019	0,3897	0,2223	0,1474	0,0444	36	100
Enterococcus	0,1942	0,0850	0,0883	0,2232	0,3068	0,2081	0,1615	0,1591	0,0424	0,0050	0,0044	0,1898	36	100
Bifidobacterium	0,0504	0,1580	0,1783	0,1812	0,0426	0,0553	0,1392	0,1806	0,0686	0,0565	0,1991	0,2857	36	100
Escherichia-Shigella	0,0383	0,0113	0,0080	0,1237	0,0422	0,0277	0,0195	0,0193	0,0280	0,0132	0,0068	0,1126	36	100
Actinomyces	0,0631	0,0102	0,0117	0,2207	0,0474	0,0175	0,0113	0,0098	0,0183	0,0002	0,0001	0,1007	33	92
Klebsiella	0,0235	0,0078	0,0061	0,0123	0,0221	0,0126	0,0092	0,0083	0,0452	0,0180	0,0111	0,0171	36	100
Anaeroglobus	0,0009	0	0	0,0103	0,0002	0	0	0	0,0028	0	0	0,0547	14	39
Clostridium sensu stricto 1	0,0204	0,0086	0,0053	0,0153	0,0382	0,0364	0,0089	0,0114	0,0032	0,0001	0,0002	0,0330	33	92
Citrobacter	0,0049	0,0022	0,0016	0,0003	0,0064	0,0047	0,0035	0,0034	0,0093	0,0052	0,0038	0,0006	35	97
Veillonella	0,0057	0,0013	0,0023	0,0548	0,0044	0,0039	0,0032	0,0025	0,0008	0	0	0,0325	30	83
Lacticaseibacillus	0,0004	0,0035	0,0056	0,0024	0,0003	0,0007	0,0039	0,0105	0,0011	0,0009	0,0028	0,0087	33	92
Ruminococcaceae - Incertae Sedis	0,0023	0	0	0,0174	0,0017	0,0003	0,0002	0,0000	0,0003	0	0	0,0153	18	50
Anaerotruncus	0,0008	6,2E-05	0	0,0128	0,0007	0	0	0	0,0003	0	0	0,0021	14	39
Lachnoclostridium	0,0005	0	0	0,0083	0,0002	3,84E-05	0	0	0	0	0	0,0063	11	31
Enterobacter	0,0003	0,0005	0,0001	0,0008	0,0002	3,84E-05	0,0002	3,22E-05	0,0005	0,0003	0,0001	0,0005	29	81
Erysipelatoclostridium	0,0008	3,1E-05	0	0,0236	0,0009	0	0	0	0	0	0	0,0139	13	36
Schleiferilactobacillus	0,0005	0,0003	0,0023	0,0052	0,0001	3,84E-05	0,0003	0,0010	0,0013	0	0	0,0105	21	58
Lactobacillales Order Unknown Genus	0,0008	0,0016	0,0032	0,0003	0,0008	0,0018	0,0053	0,0072	0,0004	0,0001	0,0003	0,0002	36	100
Enterococcaceae Family Unknown Genus	0,0003	0,0002	0,0005	0,0007	0,0003	0,0001	0,0005	0,0018	0,0002	0	0	0,0010	28	78
Flavonifractor	0,0002	0	0	0,0004	0	0	0	0	9,08E-05	0	0	0,0006	8	22
Enterobacteriaceae Family Unknown Genus	0,0003	0,0002	0,0001	0	0,0003	0,0003	0,0002	6,59E-05	0,0004	0,0005	0,0004	0,0001	28	78
Erwiniaceae Family Unknown Genus	0,0001	0	0	0	0	0	0	0	0	0	0	0,0000	3	8
Lactobacillaceae Family Unknown Genus	0,0000	0,0013	0,0023	3,62E-05	0	0,0004	0,0014	0,0016	9,73E-05	0	0,0003	0,0001	23	64
Staphylococcus	0,0003	0	0	0,0034	0,0001	0	0	0	0	0	0	0,0012	9	25
Finegoldia	0,0000	0	0	0,0003	0	3,84E-05	0	0	0	0	0	0	3	8
[Ruminococcus] gnavus group	0	0	0	0,0035	0	0	0	0	9,08E-05	0	0	0,0117	7	19
[Ruminococcus] torques group	0	0	0	0,0008	0	0	0	0	0	0	0	0,0029	6	17
Intestinibacter	0	0	0	0,0029	0	0	0	0	6,51E-05	0	0	0,0002	7	19
Parasutterella	0	0	0	0,0004	0	0	0	0	0,0001	0	0	0,0018	8	22
Lactococcus	0	0	0	0,0002	0	0	0	0	0	0	0	0	2	6
Eggerthella	0	0	0	0,0002	0	0	0	0	9,08E-05	0	0	0,00057	4	11
Erysipelatoclostridiaceae Family Unknown Genus	0	0	0	0,0003	0	0	0	0	0	0	0	0,00091	5	14
Haemophilus	0	0	0	0,0009	7,44E-05	0	0	0	0	0	0	0,00126	7	19
Rothia	0	0	0	0,0004	0	0	0	0	0	2,83E-05	0	0,00020	6	17
Varibaculum	0	0	0	0,0002	0	0	0	0	0	0	0	0	2	6
Bacteria Kingdom Unknown Genus	0	0	0	3,14E-05	3,47E-05	3,41E-05	0	0	3,03E-05	5,67E-05	1,31E-05	2,98E-05	8	22
HT002	0	0,0024	0,0005	0	0	0,0010	0,0012	0,0017	0	0	4,2E-05	0	11	31
Limosilactobacillus	0	0,0004	0	0	0	0,0003	0	0	0	0	0	0	2	6

Paeniclostridium	0	0	0	0,0003	3,72E-05	0	0	0	0	0	0	0	3	8
Aquabacterium	0	0	0	6,08E-05	6,94E-05	0	0	0	0	0	0	0	2	6
Fusobacterium	0	0	0	9,12E-05	0	0	0	0	0	0	0	0,0008	4	11
Methyloversatilis	0	0	0	0,0003	0	0	0	0	0	0	0	0	1	3
Enterobacterales Order Unknown Genus	0	0	0	0	0	7,24E-05	0	4,67E-05	0	0	1,31E-05	2,87E-05	5	14
Bacillus	0	0	0	0	0,0014	0,0005	0	0	0	0	0	0	3	8
Bacilli Class Unknown Genus	0	0	0	0	3,47E-05	0	0	0	0	0	0	3,16E-05	2	6
Clostridium sensu stricto 18	0	0	0	0	0	0	0	0	0	0	0	0,0012	3	8
Actinobacteria Class Unknown Genus	0	0	0	0	0	0	0	0	0	0	0	2,98E-05	1	3
Enhydrobacter	0	0	0	0	0	0	0	0	0	0	0	9,48E-05	1	3
**Observed Number of Genera**	**26**	**21**	**18**	**39**	**28**	**25**	**19**	**20**	**26**	**15**	**17**	**40**		

Supplementary Table S2: Differentially abundant genus level taxa as identified using Kruskal-Walli test and their average relative abundance in each treatment. F-test formula, B-base formula, BM-breast milk.

**Genus**	**T=0h**						**T=12h**						**T=24h**						**T=48h**					
	**Average RA**			**p-value**			**Average RA**			**p-value**			**Average RA**			**p-value**			**Average RA**			**p-value**		
	**Formula**	**Base**	**Breast milk**	**All**	**FvBM**	**FvB**	**Formula**	**Base**	**Breast milk**	**All**	**FvBM**	**FvB**	**Formula**	**Base**	**Breast milk**	**All**	**FvBM**	**FvB**	**Formula**	**Base**	**Breast milk**	**All**	**FvBM**	**FvB**
Actinomyces	0,063	0,047	0,018	NS	0,05	NS	0,010	0,018	0,000	NS	0,05	NS	0,012	0,011	0,000	NS	0,05	NS	0,221	0,010	0,101	0,03	0,05	0,05
Anaeroglobus	0,001	0,000	0,003	0,03	0,05	0,05	0,000	0,000	0,000	NS	NS	NS	0,000	0,000	0,000	NS	NS	NS	0,010	0,000	0,055	0,02	0,05	0,04
Anaerotruncus	0,001	0,001	0,000	NS	NS	NS	0,000	0,000	0,000	NS	NS	NS	0,000	0,000	0,000	NS	NS	NS	0,013	0,000	0,002	0,02	0,05	0,04
Bifidobacterium	0,050	0,043	0,069	NS	NS	NS	0,158	0,055	0,057	NS	0,05	0,05	0,178	0,139	0,199	NS	NS	NS	0,181	0,181	0,286	NS	0,05	NS
Citrobacter	0,005	0,006	0,009	0,03	0,05	0,05	0,002	0,005	0,005	NS	0,05	NS	0,002	0,003	0,004	NS	0,05	0,05	0,000	0,003	0,001	NS	NS	0,05
Clostridium _sensu _stricto_1	0,020	0,038	0,003	0,04	0,05	NS	0,009	0,036	0,000	0,03	0,05	0,05	0,005	0,009	0,000	0,04	0,05	NS	0,015	0,011	0,033	NS	0,05	NS
Clostridium_sensu_stricto_18	0,000	0,000	0,000	NS	NS	NS	0,000	0,000	0,000	NS	NS	NS	0,000	0,000	0,000	NS	NS	NS	0,000	0,000	0,001	0,02	0,04	NS
f_Enterobacteriaceae_g_Unknown	0,000	0,000	0,000	NS	NS	NS	0,000	0,000	0,000	NS	NS	NS	0,000	0,000	0,000	NS	0,05	NS	0,000	0,000	0,000	NS	NS	NS
f_Enterococcaceae_g_Unknown	0,000	0,000	0,000	NS	NS	NS	0,000	0,000	0,000	NS	NS	NS	0,000	0,001	0,000	NS	0,04	NS	0,001	0,002	0,001	NS	NS	NS
Enterobacter	0,000	0,000	0,001	0,03	0,05	0,05	0,000	0,000	0,000	NS	NS	0,05	0,000	0,000	0,000	NS	NS	NS	0,001	0,000	0,000	NS	NS	0,05
Enterococcus	0,194	0,307	0,042	NS	NS	NS	0,085	0,208	0,005	0,03	0,05	0,05	0,088	0,162	0,004	0,03	0,05	0,05	0,223	0,159	0,190	NS	0,05	NS
Erysipelatoclostridium	0,001	0,001	0,000	NS	0,04	NS	0,000	0,000	0,000	NS	NS	NS	0,000	0,000	0,000	NS	NS	NS	0,024	0,000	0,014	NS	NS	0,04
Escherichia-Shigella	0,038	0,042	0,028	NS	0,05	NS	0,011	0,028	0,013	0,05	0,04	NS	0,008	0,019	0,007	NS	NS	0,05	0,124	0,019	0,113	0,04	NS	0,05
Haemophilus	0,000	0,000	0,000	NS	NS	NS	0,000	0,000	0,000	NS	NS	NS	0,000	0,000	0,000	NS	NS	NS	0,001	0,000	0,001	0,02	0,05	0,04
Intestinibacter	0,000	0,000	0,000	NS	NS	NS	0,000	0,000	0,000	NS	NS	NS	0,000	0,000	0,000	NS	NS	NS	0,003	0,000	0,000	0,02	0,05	0,04
Klebsiella	0,023	0,022	0,045	NS	0,05	NS	0,008	0,013	0,018	NS	0,05	NS	0,006	0,009	0,011	NS	0,05	NS	0,012	0,008	0,017	NS	0,05	NS
o_Lactobacillales_g_Unknown	0,001	0,001	0,000	NS	NS	NS	0,002	0,002	0,000	NS	0,05	NS	0,003	0,005	0,000	NS	0,05	NS	0,000	0,007	0,000	NS	NS	0,05
Lachnoclostridium	0,001	0,000	0,000	NS	NS	NS	0,000	0,000	0,000	NS	NS	NS	0,000	0,000	0,000	NS	NS	NS	0,008	0,000	0,002	0,03	NS	0,04
Lacticaseibacillus	0,000	0,000	0,001	NS	0,05	NS	0,004	0,001	0,001	NS	0,05	NS	0,006	0,004	0,003	NS	0,05	NS	0,002	0,010	0,009	0,05	0,05	NS
f_Lactobacillaceae_g_HT002	0,000	0,000	0,000	NS	NS	NS	0,002	0,001	0,000	NS	NS	NS	0,000	0,001	0,000	NS	0,05	NS	0,000	0,002	0,000	NS	NS	NS
f_Lactobacillaceae_g_Unknown	0,000	0,000	0,000	NS	NS	NS	0,001	0,000	0,000	0,04	0,05	NS	0,002	0,001	0,000	NS	0,05	NS	0,000	0,002	0,000	NS	NS	0,05
f_Ruminococcaceae_g_Incertae Sedis	0,002	0,002	0,000	NS	0,05	NS	0,000	0,000	0,000	NS	NS	NS	0,000	0,000	0,000	NS	NS	NS	0,017	0,000	0,015	0,05	NS	0,05
Parasutterella	0,000	0,000	0,000	NS	NS	NS	0,000	0,000	0,000	NS	NS	NS	0,000	0,000	0,000	NS	NS	NS	0,000	0,000	0,044	0,02	0,05	0,04
Raoultella	0,212	0,272	0,390	0,03	0,05	0,05	0,068	0,170	0,222	0,04	0,05	0,05	0,049	0,115	0,147	NS	0,05	0,05	0,035	0,102	0,010	NS	NS	0,05

Rothia	0,000	0,000	0,000	NS	NS	NS	0,000	0,000	0,000	NS	NS	NS	0,000	0,000	0,000	NS	NS	NS	0,000	0,000	0,000	NS	NS	0,04
[Ruminococcus] _torques_group	0,000	0,000	0,000	NS	NS	NS	0,000	0,000	0,000	NS	NS	NS	0,000	0,000	0,000	NS	NS	NS	0,001	0,000	0,003	0,02	0,05	0,04
[Ruminococcus]_gnavus_group	0,000	0,000	0,000	NS	NS	NS	0,000	0,000	0,000	NS	NS	NS	0,000	0,000	0,000	NS	NS	NS	0,003	0,000	0,012	0,02	0,05	0,04
Schleiferilactobacillus	0,000	0,000	0,001	0,05	0,05	NS	0,000	0,000	0,000	NS	NS	NS	0,002	0,000	0,000	0,03	0,04	0,05	0,005	0,001	0,001	0,03	0,05	0,05
Staphylococcus	0,000	0,000	0,000	NS	NS	NS	0,000	0,000	0,000	NS	NS	NS	0,000	0,000	0,000	NS	NS	NS	0,003	0,000	0,001	0,02	0,05	0,04
Streptococcus	0,379	0,210	0,387	NS	NS	0,05	0,637	0,459	0,678	0,03	0,05	0,05	0,635	0,515	0,623	NS	NS	0,05	0,037	0,480	0,033	0,03	0,05	0,05
Veillonella	0,006	0,004	0,001	NS	0,05	NS	0,001	0,004	0,000	0,02	0,04	0,05	0,002	0,003	0,000	NS	0,04	0,04	0,055	0,002	0,033	0,03	0,05	0,05
Other	0,000	0,002	0,000	NS	NS	NS	0,000	0,001	0,000	NS	NS	NS	0,000	0,000	0,000	NS	NS	NS	0,002	0,000	0,022	NS	NS	NS
